# Transcriptome-Integrated Metabolic Modeling Identifies Candidate Metabolic Adjuvants in Antibiotic-Resistant *Pseudomonas aeruginosa*

**DOI:** 10.3390/antibiotics15080730

**Published:** 2026-07-28

**Authors:** Ceyda Kula, Rabia Cankul Kerek, Kazim Yalcin Arga

**Affiliations:** 1 Department of Bioengineering, Faculty of Engineering, Marmara University, 34854 Istanbul, Türkiye; ceyda.kula@marmara.edu.tr (C.K.); rabiacankul@marun.edu.tr (R.C.K.); 2 Health Biotechnology Joint Research and Application Center of Excellence, 34220 Istanbul, Türkiye; 3 Center for Nanotechnology and Biomaterials Applications and Research, Marmara University, 34854 Istanbul, Türkiye

**Keywords:** *Pseudomonas aeruginosa*, antimicrobial resistance, transcriptomics, genome-scale metabolic modeling, reporter metabolites, metabolic rewiring, antibiotic adjuvant candidates, biofilm

## Abstract

**Background/Objectives:** Antimicrobial resistance (AMR) poses a major global health challenge, particularly in opportunistic pathogens such as *Pseudomonas aeruginosa*. This study aimed to identify metabolic adaptations associated with antibiotic resistance by integrating transcriptomic data from drug-resistant clinical isolates with a genome-scale metabolic model (GEM) of *P. aeruginosa* under four antibiotic treatments: ceftazidime (CAZ), ciprofloxacin (CIP), meropenem (MEM), and tobramycin (TOB). **Methods:** Transcriptomic data from 414 clinical isolates were integrated with the iPau21 genome-scale metabolic model (GEM) of *P. aeruginosa.* Differential gene expression analysis was performed using DESeq2, and differentially expressed genes (DEGs) were identified using a false discovery rate (FDR)-adjusted *p*-value < 0.05 and a fold-change threshold of ≥2 or ≤0.5. Reporter metabolites (RMs) were identified using the Reporter Metabolite algorithm with an FDR-adjusted *p*-value < 0.05. Pathway enrichment analysis was performed to characterize condition-specific metabolic alterations, and pathway significance was determined using the Benjamini–Hochberg procedure with an adjusted *p*-value < 0.05. **Results:** The analysis revealed predominantly antibiotic-specific transcriptional responses, with limited overlap in DEGs across treatment conditions. Reporter metabolite and pathway enrichment analyses identified distinct metabolic adaptations associated with biofilm formation, virulence, and stress response pathways. Several metabolites, including propionic acid, acetic acid, L-inositol, glutamine, glutarate, fumarate, and melatonin, were computationally prioritized candidate metabolites for future metabolite-based adjuvant strategies aimed at enhancing antibiotic efficacy. **Conclusions:** This systems biology approach provides a comprehensive framework for identifying metabolic vulnerabilities associated with AMR in *P. aeruginosa*. The identified metabolites represent candidate antibiotic adjuvant molecules generated through computational prioritization and should be regarded as hypotheses for future experimental validation rather than validated therapeutic interventions. These findings provide a foundation for future studies exploring metabolism-based strategies to improve antibiotic efficacy and combat antimicrobial resistance.

## 1. Introduction

Antimicrobial resistance (AMR) is one of the greatest challenges to global public health, making the treatment of infectious diseases increasingly difficult. Since the introduction of antibiotics, bacteria have continuously evolved diverse resistance mechanisms, reducing the effectiveness of many first-line antimicrobial agents [1]. According to the World Health Organization (WHO), AMR was associated with approximately 1.27 million deaths worldwide in 2019, highlighting its substantial global health burden [2].

Bacterial resistance develops through several mechanisms, including modification of antibiotic targets, reduced membrane permeability, activation of efflux pumps, and production of antibiotic-inactivating enzymes [3]. Horizontal gene transfer further accelerates the spread of resistance determinants, particularly in healthcare settings where antibiotics are widely used [4]. Among multidrug-resistant pathogens, *Pseudomonas aeruginosa* is especially problematic because of its intrinsic resistance, remarkable metabolic versatility, and ability to form biofilms, all of which contribute to persistent and difficult-to-treat infections, particularly in immunocompromised patients [5,6,7,8]. Recent work has further demonstrated that the ecological adaptability, genomic diversity, and metabolic plasticity of *P. aeruginosa* are closely linked to antimicrobial resistance, virulence, and biofilm-associated persistence [9].

Previous studies have investigated antimicrobial resistance using genomics, transcriptomics, machine learning, and genome-scale metabolic modeling approaches, with each providing valuable but complementary insights into resistance mechanisms. In particular, transcriptomic studies have considerably improved our understanding of bacterial responses to antibiotic stress by identifying genes associated with resistance, virulence, biofilm formation, stress adaptation, and regulatory processes. Several recent transcriptome-based studies have further advanced our understanding of antimicrobial resistance by characterizing antibiotic-specific transcriptional responses, while computational and machine learning approaches have been employed to predict antimicrobial resistance phenotypes and identify resistance-associated biomarkers. Although these studies have demonstrated that different antibiotics induce distinct transcriptional responses, transcriptomic analyses alone do not directly reveal how resistance-associated expression patterns converge at the metabolite or pathway level. Moreover, changes in gene expression do not necessarily correspond to alterations in enzyme activity, metabolite abundance, or metabolic flux, limiting the ability of transcriptomic analyses alone to explain the metabolic basis of antimicrobial resistance [10,11].

Genome-scale metabolic models (GEMs) have become valuable systems biology tools for studying microbial metabolism and predicting cellular behavior under different environmental and antimicrobial conditions [12,13]. Previous GEM-based studies have identified essential genes, predicted metabolic flux distributions, and explored metabolic characteristics associated with bacterial growth and antimicrobial resistance. However, many of these studies have been performed without integrating condition-specific transcriptomic data or have mainly focused on flux predictions. Consequently, they provide only limited insight into the metabolic reprogramming associated with antibiotic resistance in clinical isolates [14,15]. Recent genome-scale metabolic modeling studies in *P. aeruginosa* have further demonstrated the utility of systems biology approaches for investigating virulence-associated metabolism, biofilm physiology, and metabolite-mediated potentiation of antibiotic activity. These studies highlight the growing application of metabolic network analyses in identifying mechanistic links between bacterial metabolism and antimicrobial susceptibility, while also emphasizing the need to integrate condition-specific transcriptomic information to capture resistance-associated metabolic reprogramming [14,16].

Despite these advances, an important knowledge gap remains in understanding how resistance-associated transcriptional responses are translated into metabolite- and pathway-level changes that underpin antimicrobial resistance phenotypes. Integrating transcriptomic data with GEMs provides a systems-level framework that links gene expression profiles with metabolic network organization. In particular, the Reporter Metabolite (RM) approach, first introduced by Patil and Nielsen, identifies metabolites around which coordinated transcriptional changes accumulate, thereby revealing metabolically important regions of biological networks that cannot be identified through differential gene expression analysis alone. Although originally developed as a network-based systems biology method, reporter metabolite analysis continues to be incorporated into contemporary genome-scale metabolic modeling workflows for investigating pathogen metabolism and antimicrobial susceptibility [14,17]. This integrated framework enables the identification of reporter metabolites and pathway-level metabolic rewiring associated with resistance phenotypes while facilitating the prioritization of candidate metabolic targets for further investigation [11,14].

The novelty of this study lies in integrating transcriptomic data with a genome-scale metabolic model to systematically compare antibiotic-specific and shared metabolic signatures across four clinically relevant antibiotics using a Reporter Metabolite-based framework. To achieve this, we integrated RNA-seq data from 414 clinical *P. aeruginosa* isolates with the organism-specific genome-scale metabolic model iPau21 to investigate metabolic responses associated with resistance to ceftazidime, ciprofloxacin, meropenem, and tobramycin [18]. By directly linking differential gene expression with metabolite-level network alterations, our approach enables the identification of reporter metabolites and metabolic pathways associated with resistance-related metabolic rewiring. The identified metabolites were computationally prioritized as candidate molecules for metabolite-based adjuvant strategies and represent hypotheses that require future experimental validation. Overall, this study establishes a transcriptome-integrated systems biology framework for linking transcriptomic responses to metabolic network alterations associated with antimicrobial resistance and identifies candidate metabolites for future experimental investigation.

## 2. Results

### 2.1. Transcriptional Responses Were Largely Antibiotic-Specific

**Figure 1:**
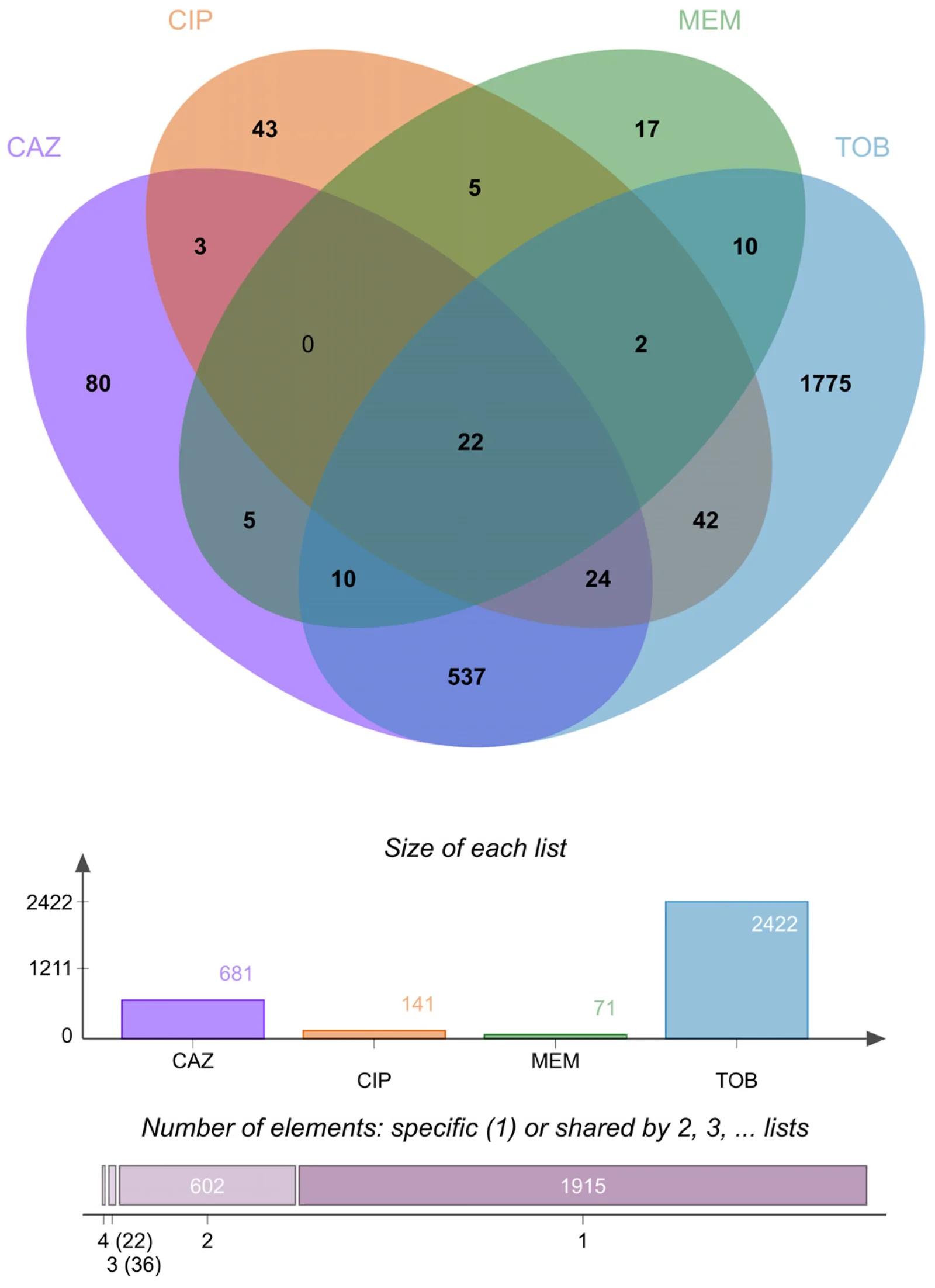
Venn diagram showing the overlap of differentially expressed genes (DEGs) identified in *P. aeruginosa* clinical isolates resistant to ceftazidime (CAZ), ciprofloxacin (CIP), meropenem (MEM), and tobramycin (TOB). The accompanying bar chart summarizes the numbers of upregulated and downregulated genes identified for each antibiotic.

Differentially expressed genes (DEGs) were identified using an FDR-adjusted *p*-value < 0.05 together with a fold-change threshold of ≥2 or ≤0.5. Based on these criteria, significant numbers of DEGs were detected in response to CAZ (*n* = 681), CIP (*n* = 141), MEM (*n* = 72), and TOB (*n* = 2422). Among the four antibiotics, TOB induced a markedly larger transcriptional response, with substantially more DEGs than the other treatment conditions. This extensive transcriptional reprogramming is consistent with the pleiotropic cellular effects of aminoglycosides, which inhibit protein synthesis and consequently activate multiple stress-response pathways affecting bacterial metabolism and cellular physiology [9,19]. Only 22 DEGs were shared across all four antibiotic conditions (Figure 1). The number of upregulated and downregulated genes also varied among antibiotics (e.g., TOB: 1252 upregulated and 1170 downregulated genes) (Table 1). Overall, these results demonstrate that each antibiotic was associated with a distinct transcriptional response. Whereas MEM and CIP resistance involved relatively limited transcriptional changes, TOB resistance was characterized by extensive transcriptome remodeling, indicating a broader adaptive response to aminoglycoside-induced stress.

Summary of the total number of DEGs identified between resistant and susceptible *P. aeruginosa* isolates for each antibiotic condition. Upregulated and downregulated gene counts are shown separately, indicating distinct transcriptional responses to ceftazidime (CAZ), ciprofloxacin (CIP), meropenem (MEM), and tobramycin (TOB).

### 2.2. Shared and Drug-Specific Reporter Metabolites

**Figure 2:**
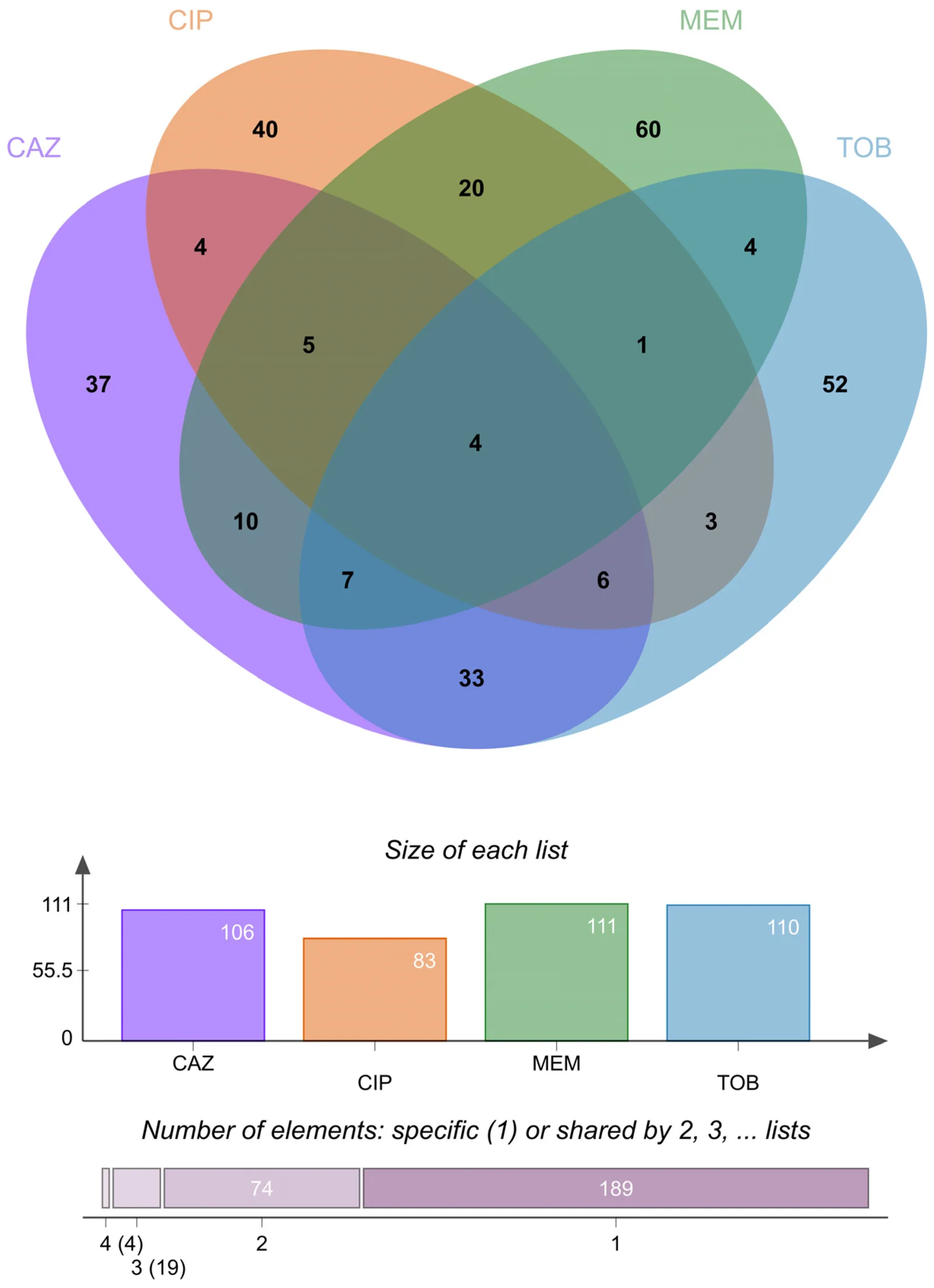
Reporter metabolites identified under different antibiotic conditions.

Reporter metabolite analysis identified 106, 83, 111, and 110 RMs in CAZ, CIP, MEM, and TOB, respectively (FDR-adjusted *p*-value < 0.05) (Online Resource S1). Only four metabolites, namely Rha-(α1,3)-N-acetylglucosamine pyrophosphorylundecaprenol (cpd17086), propionyl phosphate (cpd01844, C02876), (R)-4-phosphopantothenoyl-L-cysteine (cpd02666, C04352), and L-inositol (cpd00121, C00137), were shared across all conditions, reinforcing the notion of antibiotic-specific responses. Additionally, 37, 40, 60, and 52 metabolites were identified as drug-specific in CAZ, CIP, MEM, and TOB, respectively (Figure 2, Table 2).

RM analysis reveals condition-specific metabolites associated with resistance to ceftazidime (CAZ), ciprofloxacin (CIP), meropenem (MEM), and tobramycin (TOB).

Each color-coded section represents metabolites that show significant transcriptional perturbations with an FDR-adjusted *p*-value < 0.05 in the metabolic network surrounding differentially expressed enzymes. Only four metabolites—L-inositol, propionyl phosphate, (R)-4-phosphopantothenoyl-L-cysteine, and Rha-(α1,3)-N-acetylglucosamine pyrophosphorylundecaprenol—were common to all conditions, indicating antibiotic-specific metabolic rewiring.

List of the top ten condition-specific RMs detected under ceftazidime (CAZ), ciprofloxacin (CIP), meropenem (MEM), and tobramycin (TOB) resistance phenotypes. Metabolites are ranked according to their statistical significance (FDR-adjusted *p*-values < 0.05). KEGG and ModelSEED identifiers are provided where available. Currency metabolites were excluded to emphasize biologically relevant compounds involved in metabolic rewiring.

Among the four shared metabolites, propionyl phosphate is an intermediate involved in both the propanoate metabolism and the C5-branched dibasic acid metabolism pathways. In the latter, it is formed from 2-oxobutanoate and subsequently enters the propanoate metabolism pathway, where it is converted into propanoate. Propanoate, along with other short-chain fatty acids such as butyric and acetic acids, exhibits significant bacteriostatic effects [20].

(R)-4-phosphopantothenoyl-L-cysteine is a key metabolite in the pantothenate and coenzyme A (CoA) biosynthesis pathway. It is sequentially converted into CoA by the CoaCDE enzymes, which have been identified as biofilm-related enzymes. Inhibition of these enzymes has been predicted to enhance biofilm formation [5].

L-inositol, a polyol sugar, participates in the biosynthesis of sugars, inositol phosphate metabolism, and ABC transport processes. It has been reported that exogenous glucose supplementation enhances biofilm formation, while inositol levels decrease under hypoxic biofilm conditions. Under these conditions, the organism shifts to glucose-6-phosphate metabolism to support continued growth [21].

Rha-(α1,3)-N-acetylglucosamine (GlcNAc) pyrophosphorylundecaprenol (cpd17086) is a product of GlcNAc-diphosphate-Udp and GDP-D-rhamnose (GDP-D-Rha) reaction. It has shown that GDP-D-Rha is a rhamnose (D-Rha) donor to the GlcNAc–diphosphate–lipid acceptor in α1-3 linkage in a reaction catalyzed by putative wbpZ in the PAO1 strain [22]. This reaction builds D-Rha repeating units, which are transported from the cytoplasm to the periplasm by ABC transporters [23]. Also, it was shown that the common polysaccharide antigen (CPA) production by wbpX significantly increases the biofilm formation in PA14 [24].

### 2.3. Enrichment Analysis of Reporter Metabolites

Pathway enrichment of RMs revealed numerous drug-specific pathways (25 for CAZ, 20 for CIP, 31 for MEM, and 21 for TOB) (Online Resource S2). While 4 pathways were common to all conditions (ABC transporters, carbon metabolism, biosynthesis of amino acids, C5-branched dibasic acid metabolism), overlaps were also observed in pairs such as CAZ and MEM (e.g., arginine and proline metabolism) and CIP and MEM (e.g., valine, leucine, and isoleucine degradation) (Table 3).

Common KEGG pathways were significantly enriched (FDR-adjusted *p*-value < 0.05) among reporter metabolites identified in resistant *P. aeruginosa* isolates. The table highlights pathways shared across multiple antibiotics as well as those unique to ceftazidime (CAZ), ciprofloxacin (CIP), meropenem (MEM), or tobramycin (TOB), reflecting condition-dependent metabolic adaptations linked to biofilm formation and virulence.

**Figure 3:**
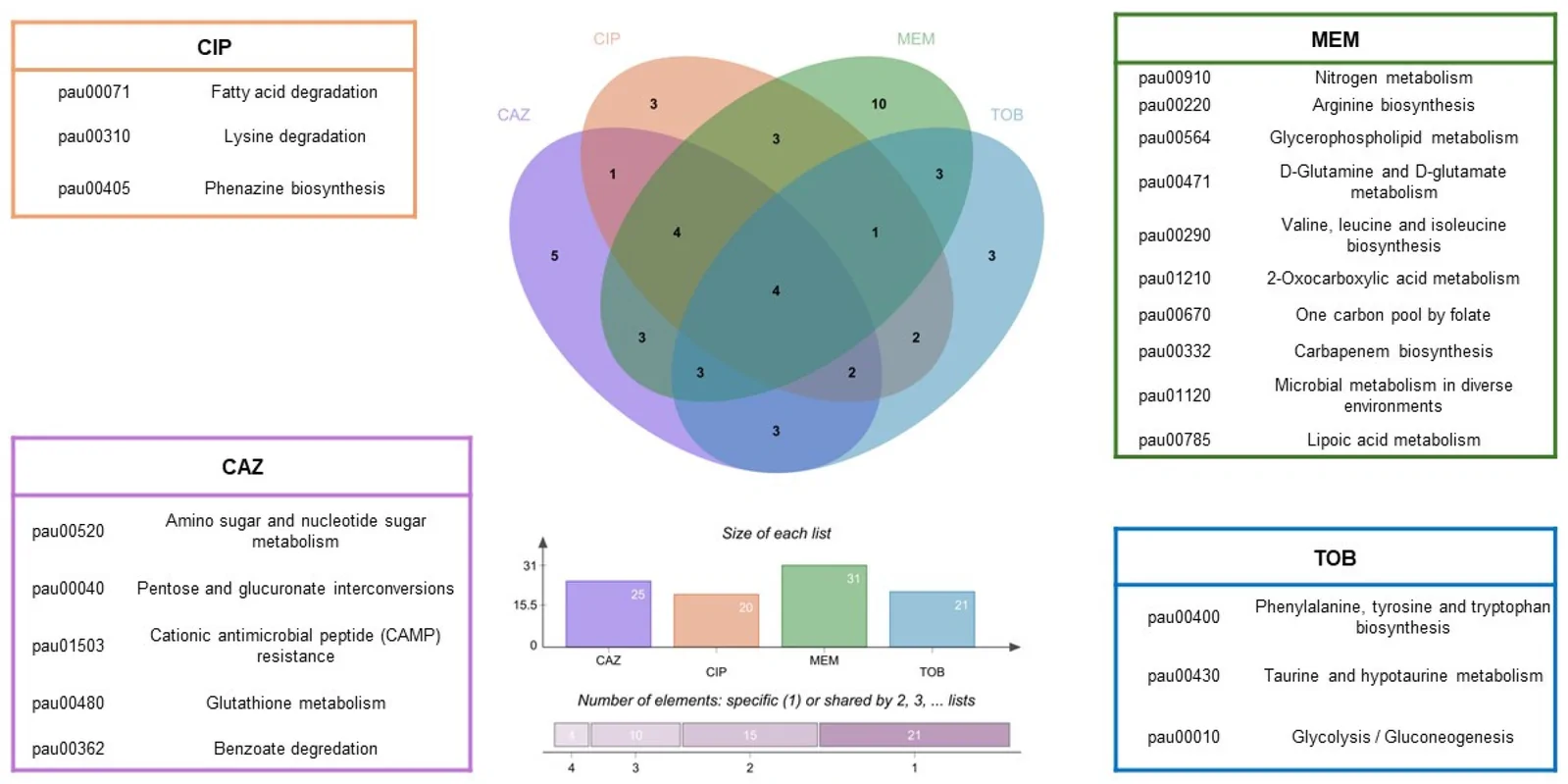
Drug-specific enriched metabolic pathways in resistant *P. aeruginosa.* Pathway enrichment analysis of reporter metabolites under ceftazidime (CAZ), ciprofloxacin (CIP), meropenem (MEM), and tobramycin (TOB) resistance phenotypes. Bars represent significantly enriched KEGG pathways (FDR-adjusted *p*-value < 0.05). Pathways common to all four conditions—ABC transporters, carbon metabolism, amino acid biosynthesis, and C5-branched dibasic acid metabolism—are marked with asterisks. Drug-specific enrichments include lysine degradation and phenazine biosynthesis (CIP), nitrogen metabolism and glycerophospholipid metabolism (MEM), and glycolysis/gluconeogenesis (TOB). Many of these pathways are associated with biofilm formation, virulence, and metabolic adaptation in *P. aeruginosa*.

Moreover, five pathways (amino sugar and nucleotide sugar metabolism, pentose and glucuronate interconversions, cationic antimicrobial peptide (CAMP) resistance, glutathione metabolism and benzoate degradation via hydroxylation) were CAZ-specific; lysine degradation, fatty acid degradation, and phenazine biosynthesis were CIP-specific; 10 pathways (nitrogen metabolism, arginine biosynthesis, glycerophospholipid metabolism, D-glutamine and D-glutamate metabolism, valine, leucine and isoleucine biosynthesis, 2-oxocarboxylic acid metabolism, one carbon pool by folate, carbapenem biosynthesis, microbial metabolism in diverse environments, and lipoic acid metabolism) were MEM-specific; and phenylalanine, tyrosine and tryptophan biosynthesis, taurine and hypo-taurine metabolism, and glycolysis/gluconeogenesis were revealed to be TOB-specific metabolic pathways (Figure 3).

Many of these pathways are known to play roles in biofilm formation and virulence in *P. aeruginosa*, including arginine and proline metabolism, glyoxylate and dicarboxylate metabolism, methane metabolism, propanoate metabolism, glycine, serine and threonine metabolism, and two-component system pathways [5]. In addition, the D-alanine metabolism pathway is known to contribute to cell stiffness [25]. Among the RMs associated with these pathways, formate and L-tryptophan were predicted to increase in concentration when biofilm-reducing reactions were inhibited. Moreover, 5-methylthio-5-deoxy-D-ribulose 1-phosphate and hydrogen sulfide, associated with cysteine and methionine metabolism, significantly altered the pathway under CIP, MEM and TOB conditions, and were predicted to decrease in concentration [5].

## 3. Discussion

The global rise in antimicrobial resistance (AMR) has been further exacerbated by the COVID-19 pandemic, which led to increased antibiotic use despite the absence of confirmed bacterial co-infections in many patients [26]. A systematic review by [27] reported a significant increase in AMR rates among patients with COVID-19. This trend has placed additional pressure on healthcare systems worldwide and highlights the urgent need for new strategies to improve the effectiveness of existing antimicrobial therapies and combat the continued emergence of antimicrobial resistance.

In situations where conventional antibiotic treatments fail, alternative strategies are needed to combat antimicrobial resistance. Among these, metabolite-based adjuvant approaches have attracted increasing attention because of their potential to enhance the efficacy of existing antibiotics [28,29]. Adjuvant compounds are administered alongside antibiotics to potentiate antimicrobial activity, interfere with bacterial resistance mechanisms, or sensitize bacteria to antibiotic treatment. For example, glutamine has been shown to enhance bacterial antibiotic uptake and reduce the viability of multidrug-resistant *P. aeruginosa* clinical isolates [30]. Such approaches may improve treatment efficacy, reduce the antibiotic doses required for bacterial clearance, and potentially lessen the selective pressure that drives the emergence of antimicrobial resistance.

In this study, we explored the molecular basis of AMR in clinical *P. aeruginosa* strains using a systems biology approach that integrates transcriptomic data with GEMs. The DEG analysis confirmed that the resistance profiles of *P. aeruginosa* were largely antibiotic-specific, with limited overlap across conditions. Notably, tobramycin resistance was associated with substantially more differentially expressed genes than the other antibiotics, suggesting a broader transcriptional response to aminoglycoside-mediated inhibition of protein synthesis. This observation is consistent with the well-established capacity of aminoglycosides to trigger widespread cellular stress responses extending beyond translational inhibition and influence multiple metabolic and regulatory processes [9,19]. These findings suggest that different antibiotics induce distinct adaptive responses, highlighting the importance of considering antibiotic-specific metabolic mechanisms associated with resistance. However, transcriptomic analyses alone provide only a partial view of bacterial adaptation. By integrating gene expression data with genome-scale metabolic modeling, our approach provided a systems-level perspective on how *P. aeruginosa* reorganizes its metabolic network in response to antibiotic stress.

RM analysis revealed predominantly antibiotic-specific metabolic responses, with only a limited number of reporter metabolites shared across the four antibiotic conditions. Notably, L-inositol was the only reporter metabolite common to all conditions, consistent with previous studies showing decreased levels during biofilm formation and increased abundance during planktonic growth of *P. aeruginosa* [21,31]. Formate and L-tryptophan were identified as reporter metabolites under TOB and CAZ treatments, respectively. Previous studies have shown that the concentrations of these metabolites increase when reactions associated with biofilm reduction are inhibited, supporting their potential involvement in biofilm-associated metabolic adaptation [5]. In addition, glutamine was identified as a reporter metabolite under both CIP and MEM conditions. This observation is particularly noteworthy because glutamine has been reported to enhance antibiotic uptake while reducing the viability of multidrug-resistant *P. aeruginosa* clinical isolates [28,29].

Furthermore, a recent metabolomics study [32] demonstrated that cadaverine and glutaric acid levels decrease significantly during biofilm formation, whereas exogenous cadaverine supplementation promotes planktonic growth and suppresses biofilm accumulation. Although cadaverine was not identified as a reporter metabolite in our analysis, glutaric acid was identified under CIP treatment. Taken together, these findings suggest that glutaric acid may also contribute to the metabolic regulation of the planktonic–biofilm transition, although this hypothesis requires experimental validation.

Several significantly enriched pathways, including glyoxylate metabolism, two-component signaling, and D-alanine metabolism, have previously been associated with *P. aeruginosa* virulence and biofilm development. Similarly, shared reporter metabolites, particularly L-inositol, are consistent with previous observations of reduced oxygen availability during biofilm formation, whereas formate, glutamine, and glutarate support earlier reports linking these metabolites to enhanced antibiotic uptake and biofilm suppression [5,21,30,31]. Furthermore, a recent untargeted metabolomics study [32] demonstrated that butanoate metabolism is upregulated, whereas arginine and proline metabolism, D-glutamine and D-glutamate metabolism, and benzoate degradation are downregulated during hypoxic biofilm development in *P. aeruginosa*. Together, these findings support the view that metabolic pathways associated with biofilm physiology represent promising candidates for future investigation and may contribute to the development of metabolite-based adjuvant strategies following appropriate experimental validation.

Enrichment analysis revealed both antibiotic-specific and shared metabolic responses. Although each antibiotic induced distinct metabolic adaptations, several common resistance-associated metabolic themes emerged across all conditions, particularly central carbon metabolism, amino acid biosynthesis, ABC transporters, and C5-branched dibasic acid metabolism. These interconnected pathways collectively illustrate how *P. aeruginosa* coordinates metabolic adaptation to support antibiotic tolerance, biofilm formation, and virulence.

ABC transporters represent one of the principal membrane-associated mechanisms contributing to antimicrobial resistance. Beyond mediating multidrug transport, they influence bacterial drug susceptibility, biofilm formation, and biofilm maturation [33,34,35]. Furthermore, their involvement in lipopolysaccharide (LPS) biosynthesis suggests an indirect contribution to antimicrobial resistance by affecting outer membrane integrity and permeability [23].

Central carbon metabolism plays a fundamental role in bacterial adaptation by redistributing metabolic fluxes in response to antibiotic stress. These metabolic adjustments regulate energy production through modulation of the tricarboxylic acid (TCA) cycle, transcription factors, and small regulatory RNAs (sRNAs), thereby promoting bacterial survival, persistence, and virulence under antimicrobial pressure [36,37,38].

Together with central carbon metabolism, C5-branched dibasic acid metabolism may contribute to resistance-associated metabolic adaptation. Amino acid biosynthesis was enriched across all antibiotic conditions, whereas C5-branched dibasic acid metabolism was associated with propionyl phosphate, which was identified as a shared reporter metabolite under all four antibiotic conditions. In addition, α-ketoglutarate (2-oxoglutarate), an intermediate of C5-branched dibasic acid metabolism, links carbon metabolism with ABC transporter-associated processes and has been implicated in regulating the pyruvate cycle, thereby influencing bacterial metabolic activity associated with antimicrobial resistance [39].

While carbohydrate metabolism and nucleotide biosynthesis were notably active under MEM treatment, CAZ treatment prominently enriched pathways involved in similar functions. In contrast, lysine degradation and phenazine metabolism, both of which have been associated with increased antibiotic tolerance during biofilm growth [40], were specifically enriched under CIP treatment. Notably, glutaric acid—a metabolite from the lysine degradation pathway—was also highlighted in our RM analysis, suggesting a potential association with the biofilm phenotype.

Several mutually enriched pathways were consistently associated with biofilm formation. These include pantothenate and CoA biosynthesis, methane metabolism, two-component systems, thiamine metabolism, propanoate metabolism, cysteine and methionine metabolism, benzoate degradation via hydroxylation, fatty acid biosynthesis, and pyrimidine metabolism, all of which have previously been linked to biofilm formation [5,41,42]. Additionally, pathways such as arginine and proline metabolism, glyoxylate and dicarboxylate metabolism, glycine, serine and threonine metabolism, and alanine, aspartate and glutamate metabolism have been associated with both virulence and biofilm development [5,43]. Collectively, these observations indicate that metabolic adaptation associated with biofilm formation represents a common feature of antimicrobial resistance across multiple antibiotic classes.

**Figure 4:**
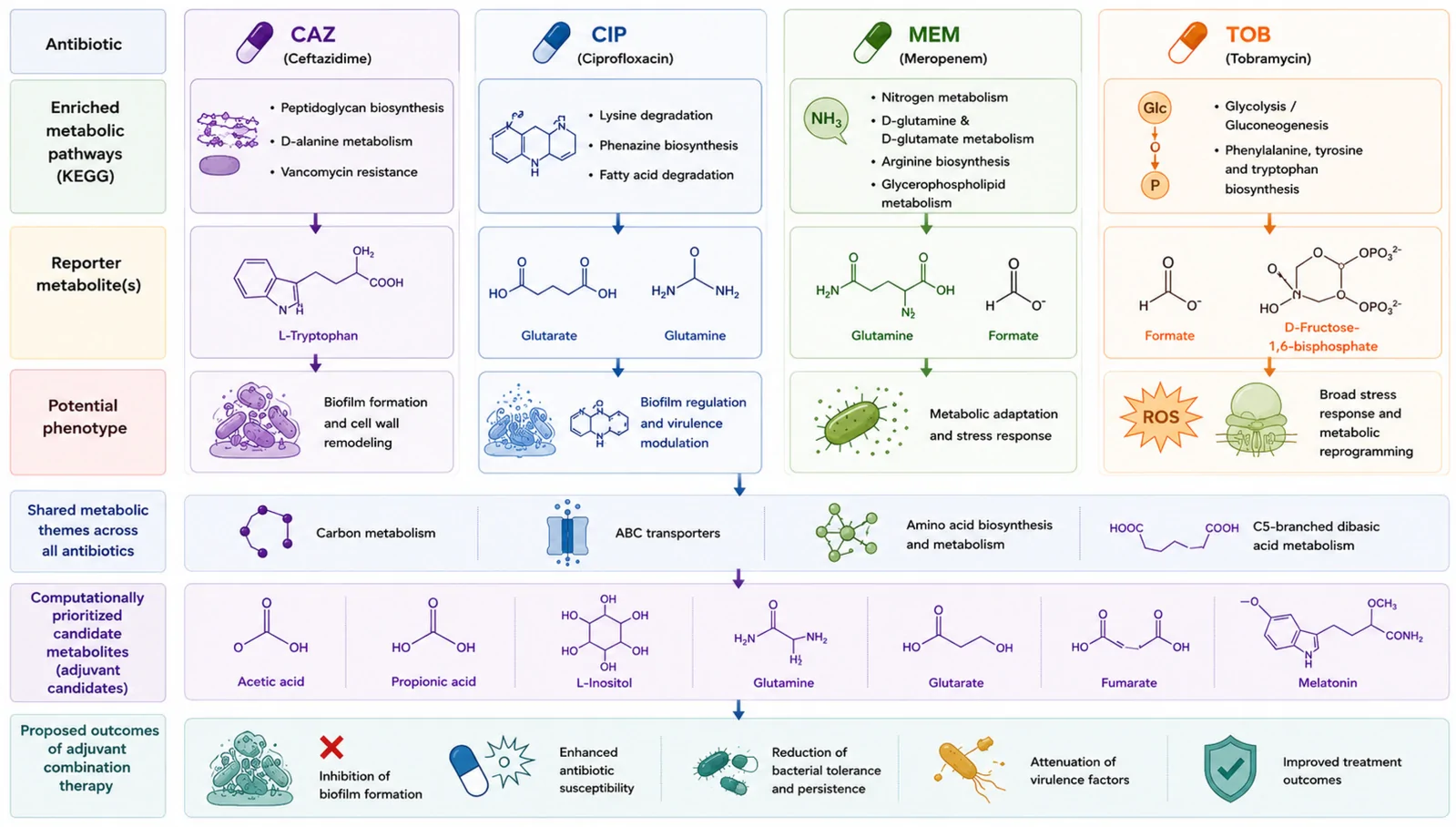
Integrated overview of antibiotic-specific metabolic rewiring associated with antimicrobial resistance in *P. aeruginosa*.

Among these pathways, citrate synthase (gltA), a key enzyme in glyoxylate and dicarboxylate metabolism, plays an important role in bacterial virulence and antibiotic tolerance. Inhibition of gltA has been shown to reduce bacterial burden, and melatonin has recently been identified as a potential inhibitor of this enzyme in Gram-negative pathogens [44,45]. Taken together, these findings further support the importance of metabolism associated with virulence and biofilm formation as a promising area for future experimental investigation. To facilitate the biological interpretation of these findings, the relationships among antibiotic-specific metabolic pathways, reporter metabolites, and their potential resistance-associated phenotypes are summarized schematically in Figure 4.

The schematic summarizes the relationships between antibiotic treatments, enriched metabolic pathways, reporter metabolites, and their potential resistance-associated phenotypes identified through transcriptome-integrated genome-scale metabolic modeling. Shared metabolic themes across all antibiotic conditions are highlighted together with computationally prioritized candidate metabolites proposed for future metabolite-based adjuvant strategies. These computationally prioritized candidates require validation through metabolomics, in vitro susceptibility and biofilm assays, in vivo studies, and ultimately clinical evaluation before therapeutic application can be inferred.

Recent genome-scale metabolic modeling studies in *P. aeruginosa* have demonstrated the value of systems biology approaches for identifying metabolic vulnerabilities, elucidating virulence-associated metabolic adaptations, and prioritizing candidate metabolites that may enhance antibiotic activity. Our findings extend these approaches by integrating transcriptomic data with reporter metabolite analysis, thereby linking differential gene expression to metabolic network organization and revealing antibiotic-specific metabolic rewiring associated with antimicrobial resistance. Together, these complementary systems biology approaches provide a robust framework for generating biologically meaningful hypotheses and prioritizing candidate metabolic targets for future experimental validation [14,16].

The pace of antimicrobial drug development continues to lag behind the rapid evolution of antimicrobial resistance. Consequently, strategies that enhance the efficacy of existing antibiotics have attracted increasing attention as complementary approaches to combat resistant pathogens [28,29,46]. In this context, metabolite-based adjuvant approaches that enhance antibiotic uptake, interfere with bacterial resistance mechanisms, or modulate bacterial metabolism represent promising areas of investigation. Among these, glutamine has been shown to enhance antibiotic uptake and improve survival in murine infection models [30]. In addition, supplementation with endogenous metabolites has been reported to enhance bacterial killing efficiency, and several studies [42,47,48] have demonstrated that combinations of metabolites or nutritional adjuvants can increase bacterial susceptibility to antimicrobial agents. Although these findings support the potential of metabolism-based adjuvant approaches, their efficacy is likely to depend on the specific pathogen, antibiotic, and metabolic context, highlighting the need for further experimental validation.

The selection of the proposed candidate metabolite set was based on an integrative prioritization strategy rather than reporter metabolite significance alone. Specifically, candidate metabolites were prioritized by considering three complementary criteria: (i) identification as significant reporter metabolites in the transcriptome-integrated metabolic analysis; (ii) previous experimental evidence supporting roles in antibiotic potentiation, biofilm inhibition, or virulence attenuation; and (iii) their participation in metabolic pathways consistently associated with antimicrobial resistance across the analyzed antibiotic conditions. Consequently, the proposed metabolite set represents a collection of biologically plausible candidate adjuvants for future experimental investigation rather than an optimized therapeutic formulation.

Using this prioritization strategy, several reporter metabolites were identified as candidate metabolites that have previously been associated with biofilm disruption, bacteriostatic activity, or modulation of bacterial metabolism. Their prioritization in the present study is based on our computational analyses, whereas the biological activities described below are supported by previously published experimental studies. These include propionic and acetic acids, which exhibit bacteriostatic effects [28]; L-inositol, which has been associated with planktonic growth and enhanced antibiotic susceptibility [29,30]; glutamine, which has been reported to enhance antibiotic uptake while reducing the viability of multidrug-resistant *P. aeruginosa* isolates [29,30]; glutarate, which may contribute to planktonic growth and suppression of biofilm formation [31]; fumarate, which has been associated with enhanced antibiotic uptake [42]; and melatonin, which has been identified as a potential inhibitor of citrate synthase, an enzyme involved in bacterial virulence [45]. Collectively, these metabolites represent computationally prioritized candidate molecules for future metabolite-based adjuvant strategies. However, these computational predictions require validation through metabolomics, in vitro susceptibility and biofilm assays, pharmacokinetic evaluation, and in vivo studies before any therapeutic application can be inferred.

This study provides a comprehensive systems biology framework for investigating antimicrobial resistance through the integration of transcriptomic data with genome-scale metabolic modeling. By linking differential gene expression with metabolic network analysis, we identified condition-specific reporter metabolites and metabolic pathways associated with resistance-related metabolic adaptation in *P. aeruginosa*. These findings provide new insights into the metabolic basis of antibiotic adaptation, particularly in the context of biofilm-associated resistance. Rather than representing validated therapeutic targets, the identified metabolites should be regarded as computationally prioritized candidates that generate hypotheses for future experimental investigation. Future studies integrating metabolomics, flux balance analysis, in vitro susceptibility and biofilm assays, pharmacokinetic evaluation, and in vivo validation will be essential to determine the biological and therapeutic relevance of these computational findings. Furthermore, the transcriptome-integrated framework presented here may be applicable to other multidrug-resistant pathogens for identifying resistance-associated metabolic vulnerabilities and supporting the future development of metabolism-based antimicrobial strategies.

## 4. Limitations and Future Directions

This study has several limitations that should be considered when interpreting the present findings. First, the identified reporter metabolites were inferred by integrating transcriptomic data with a genome-scale metabolic model and were not supported by direct metabolite concentration measurements. Consequently, future metabolomics analyses will be necessary to determine whether the predicted reporter metabolites correspond to actual intracellular metabolite levels. Second, the computationally prioritized candidate metabolites identified in this study have not yet been experimentally validated. Therefore, their effects on antibiotic susceptibility, biofilm formation, and bacterial physiology should be evaluated through in vitro susceptibility assays, biofilm assays, and subsequent in vivo studies. Third, the transcriptomic analyses performed in this study provide correlative rather than causal evidence, and changes in gene expression do not necessarily correspond to alterations in enzyme activity, metabolite abundance, or metabolic flux. In addition, this study did not include flux balance analysis, pharmacokinetic or toxicity assessments, or experimental evaluation of metabolite–antibiotic synergistic effects. Furthermore, the analyses were based on a publicly available RNA-seq dataset comprising genetically diverse clinical isolates. Because detailed phylogenetic information and strain lineage covariates were not available for all isolates, it was not possible to explicitly account for lineage effects in the present analysis. Consequently, some of the observed transcriptional differences may reflect lineage-specific genetic variation in addition to antibiotic resistance-associated responses. Although comparing resistant and susceptible isolates enables the identification of resistance-associated transcriptional patterns, the potential influence of strain heterogeneity cannot be completely excluded. Finally, the present study applied the Reporter Metabolite algorithm using its standard implementation and did not include formal sensitivity analyses to evaluate the robustness of reporter metabolite prioritization across alternative algorithmic parameters, such as the number of randomizations or neighborhood depth. Future studies should assess the influence of these parameters on reporter metabolite identification and the robustness of the resulting metabolic predictions. Future studies incorporating phylogenetic information, lineage-aware statistical models, genetically matched isolate collections, genome-informed covariates, and independent validation cohorts will be important for further disentangling lineage effects from resistance-associated metabolic adaptations.

## 5. Materials and Methods

### 5.1. Gene Expression Profiles of Clinical Strains

RNA-seq gene count data representing the transcriptomic profiles of 414 clinical *P. aeruginosa* isolates were downloaded from the NCBI Gene Expression Omnibus (GEO; accession number GSE123544). The isolates were cultured in Luria–Bertani (LB) medium and sequenced using the Illumina HiSeq 2500 platform [18]. The dataset comprised expression profiles for 6026 genes from both UCBPP-PA14 and PAO1 strains. Because the present study re-analyzed a publicly available RNA-seq dataset, RNA extraction, library preparation, sequencing quality assessment, and read-level quality filtering were performed as part of the original study [18]. The processed gene count matrix provided with the original dataset was used for all downstream analyses. Antimicrobial susceptibility profiles for ceftazidime (CAZ), ciprofloxacin (CIP), meropenem (MEM), and tobramycin (TOB) were obtained from the accompanying dataset metadata and classified as resistant, susceptible, or intermediate according to the Clinical and Laboratory Standards Institute (CLSI) minimum inhibitory concentration (MIC) breakpoints [49] (Table 4).

Classification of 414 *P. aeruginosa* clinical isolates according to their susceptibility profiles against four antibiotics—ceftazidime (CAZ), ciprofloxacin (CIP), meropenem (MEM), and tobramycin (TOB)—based on Clinical and Laboratory Standards Institute (CLSI) minimal inhibitory concentration (MIC) breakpoints. The table summarizes the number of resistant, susceptible, and intermediate strains used for differential expression analysis.

Differential gene expression (DEG) analysis was performed by comparing resistant and susceptible isolates for each antibiotic using the DESeq2 package (version 1.42.0) in R (version 4.3.2) [50]. Raw count data were normalized using the median-of-ratios method implemented in DESeq2 prior to differential expression analysis. Intermediate phenotypes were excluded from all comparisons to maximize the contrast between resistant and susceptible isolates. Differentially expressed genes were identified using an FDR-adjusted *p*-value < 0.05 and a fold-change threshold of ≥2 or ≤0.5.

### 5.2. Genome-Scale Metabolic Model of P. aeruginosa UCBPP-PA14

The genome-scale metabolic model (GEM) of *P. aeruginosa* UCBPP-PA14 (iPau21) [51] was downloaded in Systems Biology Markup Language (SBML) format from the BioModels database (accessed on 22 May 2026). The model comprises 1169 genes, 1588 reactions, and 1325 metabolites. Constraint-based metabolic analyses were performed using the COBRA Toolbox v3.1 (Constraint-Based Reconstruction and Analysis Toolbox) implemented in MATLAB R2022b (The MathWorks Inc., Natick, MA, USA).

### 5.3. Reporter Metabolite Analysis

Reporter metabolite analysis was performed using the algorithm originally described by Patil and Nielsen [17] to identify metabolites associated with coordinated transcriptional changes in neighboring enzyme-coding genes. The GEM was converted into a bipartite metabolite–enzyme network, and each metabolite was assigned a statistical score based on the differential expression of adjacent enzyme-associated genes. Reporter metabolite significance was assessed using FDR-adjusted *p*-values based on 1000 randomizations. A total of 1000 randomizations was selected to generate a stable empirical background distribution while maintaining computational feasibility, consistent with the standard implementation of the Reporter Metabolite algorithm. Metabolites with an adjusted *p*-value < 0.05 were considered statistically significant. One-reaction-layer neighborhoods were used for metabolite scoring. No formal sensitivity analyses using alternative numbers of randomizations or neighborhood depths were performed in the present study. Analyses were performed independently for the CAZ, CIP, MEM, and TOB datasets. Identified reporter metabolites were subsequently mapped to KEGG pathways for downstream pathway enrichment analysis [52].

### 5.4. Pathway Enrichment Analysis

Pathway enrichment analysis was performed using MBRole 3.0 [53] with KEGG as the reference database to identify biological pathways significantly associated with the reporter metabolites. Pathway enrichment analyses were performed separately for the reporter metabolite sets identified under each antibiotic condition. The pathway enrichment analyses included 106, 83, 111, and 110 reporter metabolites for CAZ, CIP, MEM, and TOB, respectively. Enrichment significance was evaluated using the hypergeometric test with Benjamini–Hochberg correction for multiple testing. Pathways with an FDR-adjusted *p*-value < 0.05 were considered statistically significant.

## 6. Conclusions

This study presents a transcriptome-integrated systems biology framework for investigating antimicrobial resistance in *P. aeruginosa* by combining differential gene expression analysis with genome-scale metabolic modeling and reporter metabolite analysis. The integrated approach revealed both antibiotic-specific and shared metabolic adaptations, identifying metabolic pathways associated with carbon metabolism, amino acid metabolism, ABC transporters, biofilm formation, and virulence. In addition, several reporter metabolites, including propionic acid, acetic acid, L-inositol, glutamine, glutarate, fumarate, and melatonin, were computationally prioritized as candidate metabolites for future metabolite-based adjuvant strategies aimed at improving antibiotic efficacy.

Beyond identifying individual metabolites, this study demonstrates the value of integrating transcriptomic data with genome-scale metabolic models to uncover resistance-associated metabolic rewiring that cannot be inferred from differential gene expression analysis alone. These findings provide new insights into the metabolic basis of antimicrobial resistance and establish a computational framework for identifying metabolic vulnerabilities in multidrug-resistant pathogens.

Nevertheless, the identified candidate metabolites represent computationally generated hypotheses rather than validated therapeutic interventions. Their biological relevance and potential therapeutic value require confirmation through metabolomics analyses, flux-based metabolic modeling, in vitro susceptibility and biofilm assays, pharmacokinetic evaluation, in vivo studies, and ultimately clinical evaluation where appropriate. Overall, the framework presented here provides a foundation for future experimental investigations and may facilitate the development of metabolism-based strategies to improve antibiotic efficacy against *P. aeruginosa* and other multidrug-resistant bacterial pathogens.

## Figures and Tables

**Table 1:** Differentially expressed genes (DEGs) detected under antibiotic stress.

	Treatment			
	CAZ	CIP	MEM	TOB
**Number of DEGs**	681	141	72	2422
**Number of upregulated genes**	346	72	13	1252
**Number of downregulated genes**	335	69	59	1170

**Table 2:** Top drug-specific reporter metabolites (RMs) identified in resistant strains.

Metabolite ID (ModelSEED)	Metabolite Location	KEGG ID	Metabolite	*p*-Value
**CAZ**				
cpd11567	Cytosol		14-Methyl-3-hydroxy-pentadecanoyl-ACP	2.22 × 10^−7^
cpd00136	Cytosol	C00156	4-Hydroxybenzoate	1.19 × 10^−3^
cpd11568	Cytosol		14-Methyl-trans-pentadec-2-enoyl-ACP	3.44 × 10^−3^
cpd00154	Cytosol	C00181	Xylose	5.39 × 10^−3^
cpd02172	Cytosol	C04145	Solanesyl diphosphate	6.55 × 10^−3^
cpd17088	Cytosol		Rha4glcnacppund	7.51 × 10^−3^
cpd00105	Cytosol	C00121	D-Ribose	9.84 × 10^−3^
cpd11543	Cytosol		15-Methyl-trans-hexa-dec-2-enoyl-ACP	1.12 × 10^−2^
cpd11566	Cytosol		14-Methyl-3-oxo-pentadecanoyl-ACP	1.14 × 10^−2^
cpd11551	Cytosol		6-Methyl-3-hydroxy-heptanoyl-ACP	1.41 × 10^−2^
**CIP**				
cpd11587	Cytosol	C00041, C00064	Ala-Gln	3.14 × 10^−4^
cpd02229	Cytosol	C04574	Bactoprenyl diphosphate	6.35 × 10^−4^
cpd17044	Cytosol	C21477	1-Hydroxyphenazine	1.96 × 10^−3^
cpd17077	Cytosol	C19830	Trans-2,3-dihydro-3-hydroxyanthranilic acid	4.44 × 10^−3^
cpd03187	Cytosol	C05379	Oxalosuccinate	6.13 × 10^−3^
cpd11587	Extracellular	C00041, C00064	Ala-Gln	8.68 × 10^−3^
cpd00021	Extracellular	C00023	Fe^2+^	1.23 × 10^−2^
cpd01352	Cytosol	C01968	Undecaprenol	1.32 × 10^−2^
cpd00449	Cytosol	C00579	Dihydrolipoamide	1.32 × 10^−2^
cpd00379	Cytosol	C00489	Glutarate	1.35 × 10^−2^
**MEM**				
cpd11922	Cytosol	C01651	tRNA (Thr)	1.72 × 10^−10^
cpd12229	Cytosol	C02992	L-Threonyl-tRNA (Thr)	1.72 × 10^−10^
cpd02096	Cytosol	C03284	L-3-Amino-isobutyrate	1.40 × 10^−5^
cpd11652	Cytosol	C00344	Phosphatidylglycerol	1.49 × 10^−4^
cpd23005	Cytosol	C20850	N5-hydroxy-L-ornithine	1.88 × 10^−4^
cpd00806	Cytosol	C01099	L-Fuculose1-phosphate	2.99 × 10^−4^
cpd11580	Cytosol	C00037,C00064	Gly-Gln	3.96 × 10^−4^
cpd00869	Cytosol	C01180	4-Methylthio 2-oxobutyrate	4.97 × 10^−4^
cpd00047	Extracellular	C00058	Formate	5.19 × 10^−4^
cpd11624	Cytosol	C00157	Lecithin	1.01 × 10^−3^
**TOB**				
cpd02857	Cytosol	C04691	DAHP	1.08 × 10^−3^
cpd15555	Cytosol		Phosphatidylserine—dihexadecanoyl	1.21 × 10^−3^
cpd02991	Cytosol	C04916	Phosphoribulosylformimino–AICAR–phosphate	2.19 × 10^−3^
cpd01476	Cytosol	C02191	Protoporphyrin	3.89 × 10^−3^
cpd03279	Cytosol	C05512	Deoxyinosine	4.03 × 10^−3^
cpd00810	Cytosol	C01103	Orotidylic acid	4.10 × 10^−3^
cpd11455	Cytosol	C02737	ps—BS	4.39 × 10^−3^
cpd00868	Cytosol	C01179	p-Hydroxyphenylpyruvate	4.50 × 10^−3^
cpd00290	Cytosol	C00354	D-fructose-1,6-bisphosphate	5.32 × 10^−3^
cpd00856	Cytosol	C01163	3-Carboxy-cis,cis-muconate	5.60 × 10^−3^

**Table 3:** Shared enriched metabolic pathways.

CAZ|CIP|MEM|TOB	
**pau02010**	ABC transporters
**pau01200**	Carbon metabolism
**pau01230**	Biosynthesis of amino acids
**pau00660**	C5-branched dibasic acid metabolism
**CAZ|CIP**	
**pau00460**	Cyanoamino acid metabolism
**CAZ|MEM**	
**pau00330**	Arginine and proline metabolism
**pau00053**	Ascorbate and aldarate metabolism
**pau00620**	Pyruvate metabolism
**CAZ|TOB**	
**pau00473**	D-alanine metabolism
**pau01502**	Vancomycin resistance
**pau00550**	Peptidoglycan biosynthesis
**CIP|MEM**	
**pau00280**	Valine, leucine and isoleucine degradation
**pau00640**	Propanoate metabolism
**pau00730**	Thiamine metabolism
**CIP|TOB**	
**pau04122**	Sulfur relay system
**pau00920**	Sulfur metabolism
**MEM|TOB**	
**pau00630**	Glyoxylate and dicarboxylate metabolism
**pau00061**	Fatty acid biosynthesis
**pau00680**	Methane metabolism
**CAZ|CIP|MEM**	
**pau00970**	Aminoacyl-tRNA biosynthesis
**pau00250**	Alanine, aspartate and glutamate metabolism
**pau02020**	Two-component system
**pau02030**	Bacterial chemotaxis
**CAZ|CIP|TOB**	
**pau00230**	Purine metabolism
**pau00240**	Pyrimidine metabolism
**CIP|MEM|TOB**	
**pau00270**	Cysteine and methionine metabolism
**CAZ|MEM|TOB**	
**pau00770**	Pantothenate and CoA biosynthesis
**pau00260**	Glycine, serine and threonine metabolism
**pau01240**	Biosynthesis of cofactors

**Table 4:** Phenotypic characterization of strains for susceptibility or resistance to antibiotics.

Number of Strains/Antibiotics	CAZ	CIP	MEM	TOB
**Resistant strains**	165	199	244	130
**Susceptible strains**	169	159	110	276
**Intermediate**	80	56	60	8

## Data Availability

The RNA-seq datasets analyzed during the current study are available in the NCBI Gene Expression Omnibus repository, with accession number GSE123544 (https://www.ncbi.nlm.nih.gov/geo, accessed on 24 July 2026). The genome-scale metabolic model of *P. aeruginosa* UCBPP-PA14 (iPau21) is available in the BioModels database (https://www.ebi.ac.uk/biomodels, accessed on 24 July 2026).

## Supplementary Materials

The following supporting information can be downloaded at: https://www.mdpi.com/article/10.3390/antibiotics15080730/s1, Online Resource S1: Reporter Metabolite Analysis Results for Each Drug (FDR-adjusted *p*-value < 0.05). Online Resource S2: Pathway Enrichment Analysis Results for Each Drug (Kegg Pathways).

## Abbreviations

The following abbreviations are used in this manuscript:

ABC	ATP-binding cassette
AMR	antimicrobial resistance
CAZ	ceftazidime
CAMP	cationic antimicrobial peptide
CIP	ciprofloxacin
CLSI	Clinical and Laboratory Standards Institute
CoA	coenzyme A
CPA	common polysaccharide antigen
DEG	differentially expressed gene
DGE	differential gene expression
FDR	false discovery rate
GEM	genome-scale metabolic model
GlcNAc	N-acetylglucosamine
KEGG	Kyoto Encyclopedia of Genes and Genomes
LB	Luria–Bertani
LPS	lipopolysaccharide
MBRole	Metabolite Biological Role
MEM	meropenem
MIC	minimum inhibitory concentration
NCBI	National Center for Biotechnology Information
PA14	UCBPP-PA14 strain of *Pseudomonas aeruginosa*
PAO1	*Pseudomonas aeruginosa* PAO1 strain
RM	reporter metabolite
RNA-seq	RNA sequencing
SBML	Systems Biology Markup Language
sRNA	small RNA
TCA	tricarboxylic acid
TOB	tobramycin
WHO	World Health Organization
